# Optimal reactive power compensation in electrical distribution systems with distributed resources. Review

**DOI:** 10.1016/j.heliyon.2018.e00746

**Published:** 2018-08-22

**Authors:** A. Águila Téllez, G. López, I. Isaac, J.W. González

**Affiliations:** a Universidad Politécnica Salesiana, Quito, Ecuador; b Universidad Pontificia Bolivariana, Medellín, Colombia

**Keywords:** Energy, Electrical engineering

## Abstract

*In this paper an exhaustive bibliographical revision of the mathematical methods used for the optimal selection and location of reactive power compensating elements is developed*, the results obtained by different authors for different objective functions are analyzed and a scientific problem in the conflict that the electric variables show when analyzed individually is identified; thus demonstrating the need to analyze this problem in a multi-criteria way and taking into account topologies of distribution grids with distributed generation and energy storage. This research demonstrates that reactive power compensation in distribution grids with distributed resources is a problem that must be analyzed from multiple criteria that consider several objective functions to be optimized; thus achieving a global solution that contemplates an optimal location and dimensioning of reactive power compensating elements that contribute to the joint improvement of the voltage profiles, minimization of power losses, harmonic mitigation, increased line capacity, voltage stability and power factor improvement, all of them to a minimum investment cost. A theoretical heuristic is also proposed to solve the described problem, based on the multicriteria optimization method.

## 1 Introduction

The main objective of electricity distribution grids is to transport electric energy to end users with required standards of efficiency, quality and reliability, which requires minimizing energy losses and improving transport processes [1]. Reactive power compensation is one of the well-recognized methods for its contribution to the reduction of energy losses, along with other benefits; Such as power factor correction, increase of the transport and operation capacity of lines and devices of the grid, voltage stability and improvement of the voltage profile, all of them subject to different operating restrictions [2, 3, 4, 5]. The proper integrated control of the reactive power flows and of the voltage profile in distribution grids has become a very serious problem of complex solution, due to the characteristics of the distribution grids. In this paper a state of the art based on a large bibliographical review will be developed to demonstrate that the great majority of authors, who have done research to solve problems of reactive power compensation, have proposed solutions to a single objective function, either to minimize power losses, to improve power factor, to release capacity in lines and equipment, to improve voltage profiles, to guarantee voltage stability, to mitigate harmonics, among others [6, 7, 8, 9, 10]. For this, many heuristic and metaheuristic methods have been applied and described, which are based mainly on exploratory searches to find this type of solution that lies in the location and dimensioning of compensating elements within a distribution grid. The purpose of this research is to demonstrate the need to respond in a global and efficient way to the control of the electric variables affected by the reactive power flows demanded by the loads in the electric distribution systems with predominantly inductive nature. The importance of focusing this analysis on distribution grids with distributed resources will also be highlighted, because in the near future the distribution grids are destined to be self-sustaining grids with renewable generation of non-polluting and non-extinguishable sources. This type of micro-grid topologies that can be isolated requires a particular analysis in the reactive power compensation due to the bidirectional power flows that exist in these grids.

The main objective of this work is to identify the variation that can have a compensation solution associated to the optimal location and dimensioning of compensating elements in a distribution grid with distributed resources, when it is not analyzed in a multicriteria way. It will be analyzed how the different authors offer different solutions in the location and dimensioning of reactive power compensating elements for different objective functions; this problem holds that an optimal solution for a single objective function can conflict with the solutions to the other objective functions. In addition, the analysis in real micro-grids with distributed resources incorporates a complexity to the problem due to the own compensation of generators, which in the case of solar photovoltaic generators, in most cases, compensate only for active power, which deteriorates enormously the power factor of the grid [11, 12].

**Fig. 1:**
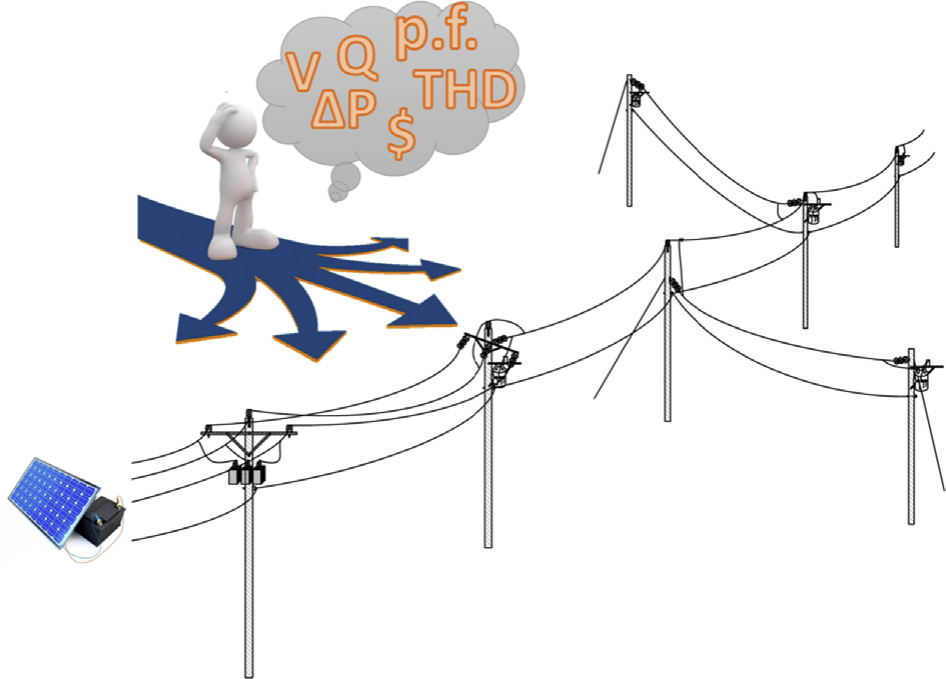
Graphical representation of the scientific problem for the compensation of reactive power in distribution grids.

The contribution of this research is associated with identifying a problem in the solutions proposed by many authors to compensate reactive power with a single objective function and with demonstrating the conflict that exists between the variables when being analyzed individually, which grounds the need to analyze this phenomenon in a multicriteria way and propose an optimal solution to the set of variables that are affected by the location of compensating elements in distribution grids with distributed resources, which encompasses a more real study scenario in the current grids. A broad theoretical and conceptual description is also detailed and a compensation decision method based on multiple criteria is proposed. Fig. 1 shows a graphical representation of the proposed scientific problem, in the grid topology with distributed resources.

The organization of the work is as follows: Section 2 (Main text) explains the main characteristics of the variables analyzed in the investigation and the impact on the distribution grid, discusses the analysis of the bibliographic review and identifies the scientific problem. Section 2 also shows the mathematical model proposed for the theoretical solution to the problem and describes future work. Finally, conclusions can be found in Section 3.

## 2 Main text

### 2.1 Analysis of the main variables that intervene in the problem of reactive compensation in distribution grids

The electricity distribution grids in the medium voltage are in charge of transporting energy from substations of Sub-transmission to distribution transformers. These circuits have particular characteristics and must comply with a series of technical requirements to keep processes in efficient parameters and provide a high quality service [13, 14, 15, 16, 17]. The global analysis of the transport of electrical energy with efficiency and quality in distribution grids is a complex process that depends on multiple criteria, because these systems present different types of grid topologies, different construction and configuration characteristics, multiple connections, loads of different natures, lines without transpositions, many points of union or splices in coexistence with the flora and the fauna.

### 2.2 Loss of power and energy in distribution grids

The losses of power and energy in distribution grids are mostly associated with the conversion of electric energy to heat subject to the circulation of currents by the electric conductors, a phenomenon described as a Joule effect [18].

Active power losses are given by:

(1) $$\Delta P=\sum_{i=1}^{n}I_{i}^{2}*R_{i},$$

Where: n is the number of nodes in the system, $I_{i}$ is the current value at the node i and $R_{i}$ is the resistance at the node i [18]. The distribution circuits, in spite of the typical nature of their loads, are predominantly inductive due to their short distances and medium voltage levels, predominating the aerial type of construction [14]. Therefore, the loads associated to each of the distribution transformers demand a reactive power consumption to be able to generate the inductive flows without generating useful work with the consumption of this type of powers. This reactive power in three-phase systems for a given node is given by:

(2) $$Q_{i}=\sqrt{3}*V_{i}*I_{i}*\sin{Ø}_{i}.$$

Where $V_{i}$ e $I_{i}$ are the voltage and current at the node i, ${Ø}_{i}\;$ is the angle between voltage and current at the node i.

In non-compensated distribution systems, the reactive power flows are consumed in the grid and the reactive components of the currents that demand these inductive loads normally circulate throughout the distribution circuit, causing high losses due to the Joule effect previously described. For this reason, the reactive power compensation, subject to the proper selection and location of compensating devices, is of great importance in minimizing losses of power and energy. The location of compensating devices allows the delivery of reactive power flows to the grid, thus preventing them from being delivered by the grid with undesirable values in the circulating currents [1].

### 2.3 Correction and improvement of the power factor

The power factor is basically defined as the relation between the active power in (W) and the apparent power given in (VA) [19]. The power factor can be calculated in a three-phase or single-phase system as:

(3) $$\text{PF}=\cos{Ø}_{i}=\frac{P_{i}}{S_{i}}=\frac{P_{i}}{V_{i}*I_{i}},$$

Where: PF and cos ${Ø}_{i}\;$ are the recognized basic symbols for naming the power factor [20], $P_{i}$ is the active power or real power at the node i, $S_{i}$ is the apparent power at the node i and $V_{i}\;e\;I_{i}$ are the voltages and currents at the node i.

The correction of the power factor to desired values close to 1 (ideal case) is an improvement that all the distribution companies seek to implement, as well as industrial users that do not meet the minimum requirements on the efficient use of electric energy and therefore, they are penalized [21].

The distribution grids in medium voltage (MV) transport energy to the distribution transformers which in most cases, feed predominantly inductive loads; this deteriorates the power factor on a large scale, so it is necessary to implement compensation measures of the reactive power in these grids to reduce the consumption of reactives by minimizing the difference between the active and apparent power to improve the power factor. Improving the power factor implies a reduction of energy costs, release of the electrical capacity of the distribution system and improvement of the voltage levels [22, 23].

### 2.4 Improvement of voltage profiles

Ensuring the reliability and stability of medium voltage distribution grids is one of the biggest challenges for energy distribution companies, since energy must reach end users with quality standards that demand constant improvement to maintain the levels of stable voltages within the parameters governed by the standards established in each country for the different voltage levels [24]. The improvement of the voltage profiles in distribution grids, seeking to increase stability and reliability, has been achieved through the insertion of distributed generation, variation of transformer TAPs, voltage regulators, capacitor banks or static reactive power compensators, SVC by its acronym in English, among others [15, 24, 25].

Static reactive power compensators can maintain a pre-programmed stable voltage level. If the voltage in the connected node is high, the compensator works in an inductive zone and consumes reactive power of the load, this can happen in hours of the dawn when the load demand lowers and if, on the contrary, the voltage in the node is low (peak demand times) [26] the compensator works in a capacitive zone and releases reactive power functioning as a generator, and in this way, it keeps the distribution system stable. This same effect can be achieved with the use of voltage regulators or with the variation in the TAP derivations of transformers, which can regulate the transformation process in different voltage transformation relations, either to reduce or to increase delivered voltage levels, guaranteeing the stability of the system [27].

### 2.5 Harmonic mitigation

Among the static power reactive power compensator devices based on power electronics, the SVCs (previously described) stand out, which contain capacitance steps in parallel with reactances, both programmed by an automatic control system that decides whether the SVC should behave as a reactive generator and raise the system voltage, or behave as a load and absorb reactive from the grid by stabilizing the voltage levels to set parameters [28, 29, 30]. These devices inject a considerable harmonic component that must be taken into account in the global analysis of the problem of reactive power compensation, since it is a variable that conflicts with the purpose of optimization of reactive power flows. It must be ensured that the limits of total harmonic distortion of current and voltage do not exceed the values established by the norms of energy quality [28, 31, 32].

The Total Harmonic Distortion Rate (THD) can be calculated as shown below [28, 31, 32].

(4) $$THD\%=100*\frac{\sqrt{\sum_{i=1}^{H}{\left(V_{i},h\right)}^{2}}}{V_{i},1},$$

Where:

$V_{i},h$ is the voltage component corresponding to the harmonic h at the node i.
$V_{i},1$ Is the fundamental voltage component (1st harmonic) at the node i.
H is the maximum harmonic order to be taken into account in the calculation.

### 2.6 Cost analysis of reactive power compensation devices

All the improvements associated with reactive power compensation in distribution grids have an investment and maintenance cost, which must be analyzed together with the gains from energy loss reduction concepts provided by the compensating devices; in addition to the benefits of quality and reliability, which are also qualitative goals that are sought with the use of these devices.

### 2.7 Distribution grids with distributed generation

Distributed Generation (DG) is a technology that provides added value of active power to power electrical systems. The location of this type of technology is usually implemented as close as possible to end users or important loads requiring a higher degree of reliability and greater stability in the voltage supplied. Among the different types of sources of distributed generation of renewable energies, the most used in distribution grids are wind and photovoltaic, although in many cases other types of sources can be evidenced [2]. These types of DG sources must be carefully evaluated to determine, depending on the topology and location, the best possible together with other important factors such as installed capacity and their location on the grid. The latter is of vital importance since an inadequate location can contribute to the unwanted injection of active and reactive power flows that could increase energy losses in the system and generate overvoltages in the two near the DG, in addition to high costs without achieving the proposed objectives [33, 34, 35]. The DG offers great benefits in the efficiency, stability and reliability of the distribution systems, especially in radial grids that travel long distances in which a small increase in the load capacity can destabilize the system with high disturbances and voltage drops. The compensation with DG with optimal location in these end nodes can eliminate that overload of the line and also restore the required values of voltage [25, 36]. It also helps to assume an increase of the existing load in possible future scenarios, even with extension of the distribution grid, maintaining stable levels of voltage, losses and capacity of the grid. [4]. Due to this, we can establish the DG as a compensation element within the distribution systems [17, 27, 28, 29, 30].

### 2.8 Energy storage in distribution grids

One of the most novel and desired goals for the transition from traditional electrical systems to Smart Grids is the incorporation of energy storage. The storage of energy in the distribution grids contributes in a remarkable way to raise the efficiency, quality and reliability of these systems, offering high advantages against fluctuations and allowing to control with greater flexibility the frequency and the voltage in the systems of distribution [41, 42, 43]. The direct coupling to the grid of energy storage banks is an issue that has been gaining acceptance as a source of distributed generation together with all the sophisticated control and communication elements for the proper use of this technology [44]. In radial distribution grids with DG, it is often very expensive at the end nodes to deliver energy from the system to the power supply, since the transport of this energy causes considerable Joule losses. Therefore, in these particular cases it is a very efficient option to place energy storage near the DG in order to store and reuse the generated energy, at times of system instability [45]. In the same way, there are systems of micro-grids with hybrid energy storage, using in addition to the banks of batteries, capacitors that allow the loading and unloading depending on the operation of the grid in active and reactive power [46].

### 2.9 Bibliographical review

For the analysis of the treatment of the variables involved in the problem of optimization of reactive power flows, an exhaustive bibliographical revision is made taking into account many virtual libraries, including IEEE Xplore, ScienceDirect, Scopus, among others. This bibliographic review aims at comparing the intelligent optimization techniques treated by the different authors to solve the compensation problems and with this result to establish comparisons between the different multiobjective proposals according to the distribution grid scenarios considered, and the number of variables which intervene in each of the proposed mathematical models. The bibliographic review contains the most current and novel articles in the subject matter and they are mentioned in the following references:

Scientific articles analyzed: [6, 7, 10, 23, 27, 29, 30, 37, 38, 39, 40, 47, 48, 49, 50, 51, 52, 53, 54, 55, 56, 57, 58, 59, 60, 61, 62, 63, 64, 65, 66, 67, 68, 69, 70, 71, 72, 73, 74, 75, 76, 77, 78, 79, 80, 81, 82, 83, 84, 85, 86, 87, 88, 89, 90, 91, 92, 93, 94, 95, 96, 97, 98].

**Fig. 2:**
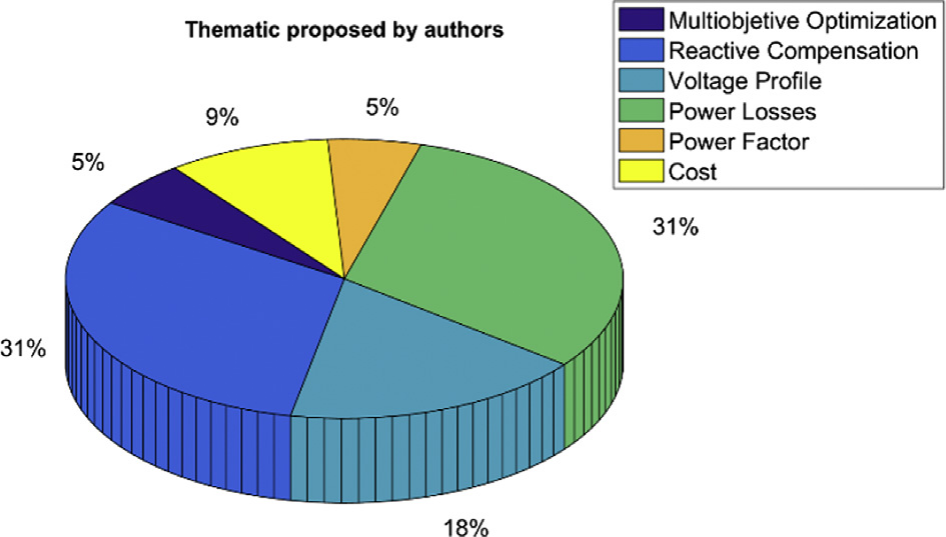
Graphical representation of thematic treatments for reactive power compensation in distribution grids.

Fig. 2 shows the treatment given by the authors, according to the subject matter, to solve the problem of compensation of reactives in distribution grids based on the optimization of reactive power flows. It can be seen that the most considered variable is: power losses.

**Fig. 3:**
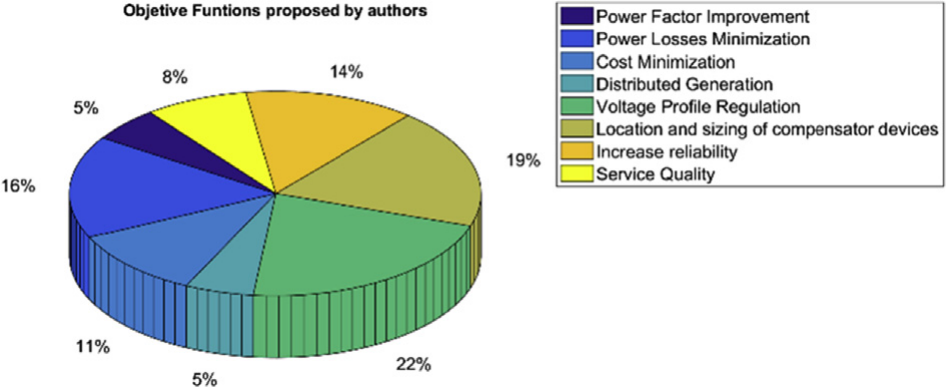
Graphical representation of problem formulations for reactive power compensation in distribution grids.

Fig. 3 shows the treatment given by the authors to the different objective functions in the problem approach. Taking into account the bibliographic review, we were able to establish these metrics given in Fig. 5, showing the percentage of study that each of the objective functions involved in the reactive power compensation has had. It can be seen that, for the case of the proposed objectives, the variable with the greatest application in this problem has been “Regulation or improvement of the voltage profiles”.

**Fig. 4:**
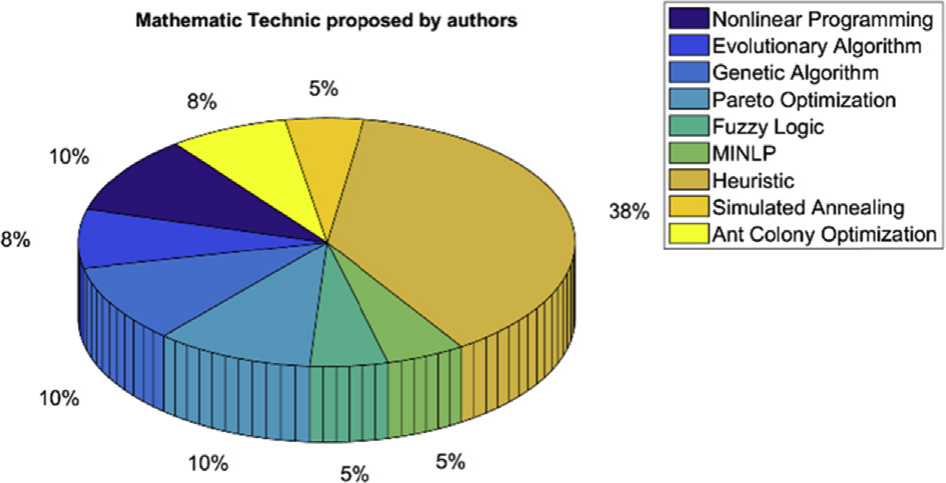
Graphical representation of the mathematical techniques used by the authors for the compensation of reactive power in distribution grids.

Fig. 4 shows the majority of mathematical techniques proposed by the authors for the problem of compensation of reactives in distribution grids, and the percentage analysis of the use of each of these techniques in the articles reviewed. Then, it can be concluded that heuristic techniques are the most used to solve this type of problems of high complexity, although in most articles the authors propose heuristic techniques that have their support in other mathematical techniques.

With the results obtained in this bibliographic review, we were able to identify the problem of a mathematical model that contemplates the joint analysis of all the variables involved in the problem of optimization of reactive power flows. We can also say that most of the proposed models consider distributive scenarios without distributed resources, which is far from the reality of power systems in general, which are currently immersed in many micro-networking projects with the incorporation of distributed resources.

The tendency of the new mathematical proposals by the authors in the publications from 2016 has been to expand the area of exploration in the variables assumed and the scenarios treated to find the optimal location and the dimensioning of compensating devices of reactives in distribution grids with distributed resources. This demonstrates the importance and interest in the subject; however, in the bibliographic review we could not find a proposal that contemplates the optimization of reactive power flows in distribution grids with distributed resources, and that considers all the variables that can be modified with the reactive power flows.

From a selection of many articles, we chose 16 articles with greater relevance and scope to the problem identified in this research. This bibliographical review is shown in Table 1, indicating the articles with a greater approximation to the real problem and that are updated in the discussion of this problem.

**Table 1:** Bibliographic review.

	Analyzed papers		Treatment of objective functions					DSTATCOM	Type of compensation			
	Paper	Publication year	Cost optimization	Minimization of losses	Stability and voltage profile	Improvement of PF.	Harmonic reduction		Capacitor banks	SVC	Energy storage	Distributed generation
1	[52]	2016	X	X	X	X					X	X
2	[67]	2018	X	X	X							
3	[66]	2018			X				X		X	X
4	[74]	2017		X					X		X	X
5	[75]	2017	X	X	X			X			X	X
6	[94]	2016		X	X			X			X	X
7	[93]	2016	X	X	X	X			X			
8	[50]	2016	X	X	X							X
9	[53]	2016	X	X	X						X	X
10	[49]	2016		X	X	X			X			
11	[48]	2016	X	X	X				X			
12	[27]	2016	X	X	X				X			X
13	[39]	2015		X	X				X			X
14	[30]	2015	X	X	X					X		
15	[37]	2015	X	X	X							X
16	[100]	2014			X		X				X	X

It may be noted that in [52] is where a more complete analysis is obtained. However, it does not analyze all the variables nor the compensation through compensating devices; compensation is raised from distributed resources. From the selected articles, it can be evidenced that only one of analyzed papers takes into account the harmonic mitigation, but it is important to clarify that in many other articles this variable is considered as an objective function in the reactive power compensation, mainly through compensation devices based on power electronics, which introduce a large amount of harmonic component to the grid that must be taken into account for this type of global analysis [62, 99].

### 2.10 Identification of the problem

In Section 2 we analyzed the different variables and topologies of distribution grids that can be affected and which can influence in the decision making for the optimal selection and location of compensating elements in a distribution grid, with the objective of compensating multicriterially the flows of reactive power. In this section we will analyze some results found by different authors and we will also compare the incidences in the rest of variables when a solution is presented to a single objective function.

To solve the problem of optimum selection, location and dimensioning of reactive power compensation devices in distribution grids, many mathematical models based on heuristics and meta-heuristics have been described and developed, which function as search algorithms by doing a scan on the nodes and lines of the system under study. They could be real cases or typical distribution systems of the IEEE. Among the most implemented algorithms to solve this type of problems, we can mention the Simulated Annealing, Tabu Search, Genetic Algorithm, Ant Colony Optimization, Particle Swarm Optimization, Mixed Integer Nonlinear Programming, among many others. For the case of the analysis that identifies the problem of variable conflict, the Simulated Annealing algorithm will be implemented, which is a meta-heuristic of probabilities that looks for an optimal combinatorial solution from an estimate for a global optimal solution of an objective function in a specific search area. This begins with a group of arbitrary chains that have a configuration of power with the installation of reactives of the population generated from an initial temperature. For this reason, the approach can generate a series of different ways of searching, trying to locate the best solutions that finally come in a global optimum, if it exists. [101].

In this chapter, a case study of a typical 30-bar IEEE circuit will be analyzed, where a Simulated Annealing (SA) algorithm will be implemented to find the optimal location and dimensioning of capacitor banks to compensate for reactive power with a case 1, which will aim at minimizing total power losses in the study circuit. Then, the algorithm (SA) will be implemented in the same IEEE system of 30 bars, with a case 2 that will aim at improving the voltage profiles, optimally bringing them closer to 1 per unit. Finally, the results will be analyzed in each case, looking for the margin of conflict that each variable suffered with the location and the obtained sizing. Both cases will be analyzed in a scenario with maximum location restriction of two banks of capacitors and maximum capacity of 15 MVAR. This restriction aims at limiting the cost variable and making the algorithm, in both cases, seek to compensate the system up to this maximum value in both cases equally.

Case 1: The behavior of the voltage profiles in a typical 30-bar IEEE circuit will be analyzed, which was compensated by the location of two capacitor banks with an objective function of minimum power loss. The IEEE 30-bus system is composed of 6 generators at the nodes [1 2 5 8 11 13], 4 transformers at the nodes [11 12 15 36], 20 loads and a total of 41 lines.

Case 2: The behavior of power losses in a typical 30-bar IEEE circuit will be analyzed, which was compensated by the location of two capacitor banks with the objective of improving the voltage profiles in the same circuit described in case 1.

**Fig. 5:**
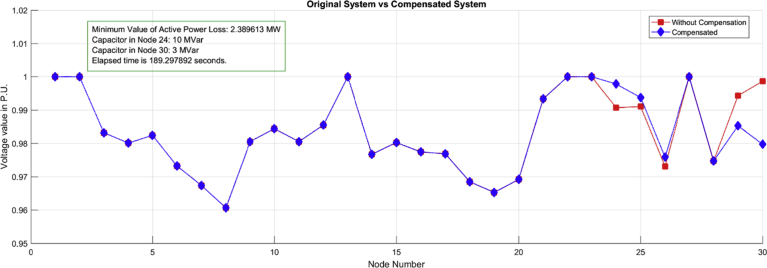
Case 1: Optimal selection and location of capacitor bank for power loss minimization.

**Fig. 6:**
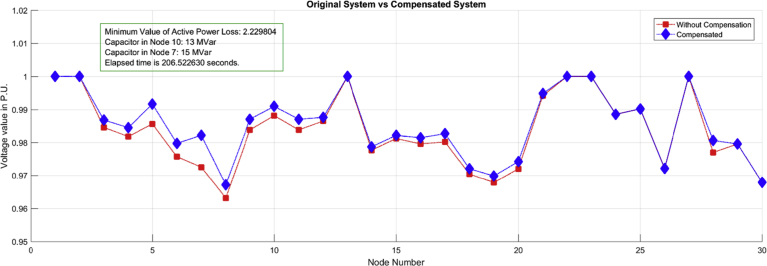
Case 2: Optimal selection and location of capacitor banks for the improvement of voltage profiles.

When comparing the results of each case shown in Figs. 5 and 6 respectively, it can be shown that for each objective function the algorithm chose different capacities of capacitor banks located in different nodes for the same circuit, which demonstrates the theory previously raised. We can further appreciate in Fig. 5 that for this case with the optimal selection and location of capacitor banks chosen by the algorithm, the power losses were reduced to a desired minimum value. However, voltage profiles were not corrected, even at nodes 29 and 30 the system shows a worsening of the voltage quality with respect to the base case, even with the location of a compensation at node 30, which indicates problems with the stability of voltage when the relation between reactive power variation and voltage variation becomes negative. Likewise in Fig. 6 (Case 2) it can be seen that the power losses could not be reduced to the minimum value that is achieved in case 2. However, voltage profiles were corrected at most nodes with the location of two capacitor banks, 13 MVAR at node 10 and 15 MVAR at node 7, locations and sizing different from those found in the case 1 by the same algorithm, using the same analysis circuit and using the same restrictions.

With this small comparison it was shown that finding an optimal solution to the problem of compensation of reactive power with a single objective function, does not provide encouraging results for the rest of variables that can be affected by the problems; for this reason we can state that it is quite important to solve this problem considering all the criteria that define the variables that can be affected with the reactive power flows.

**Fig. 7:**
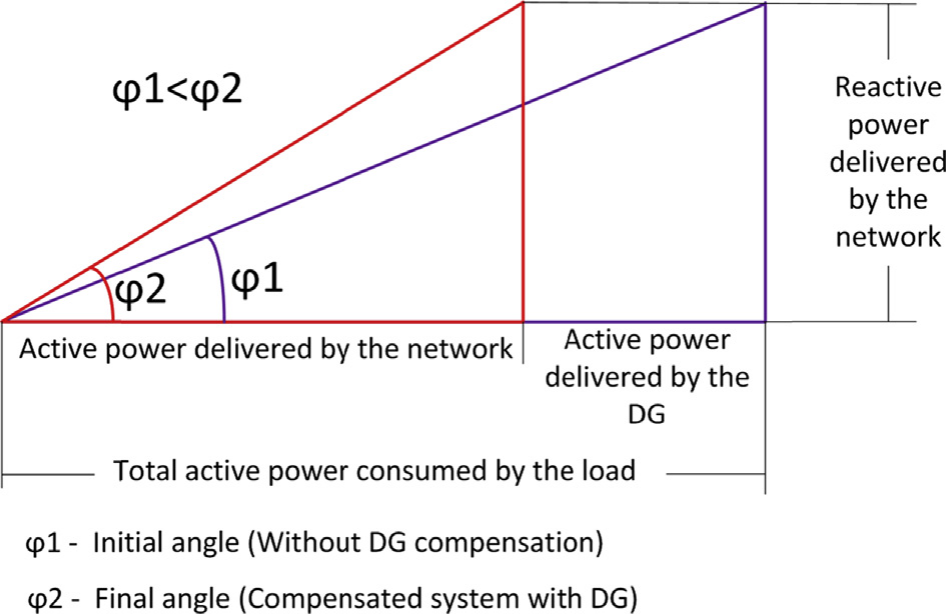
Graphical representation of the deterioration of the power factor with active power compensation only.

If this same problem is analyzed in a grid with distributed resources, or at least compensated in addition with distributed solar photovoltaic generation, the system can be affected by a low power factor, since this variable conflict with the active power compensation only, as shown in Fig. 7.

Assuming that the angle of the power factor: $Ø=\tan^{-1}\frac{Q}{P}$, and PF=cosØ, with an increase of only active power compensation while the reactive power delivered by the grid remains almost constant. The electric power trading company would be seeing an increase in the angle of the power factor, which leads to a decrease in the power factor (PF). This is because the distribution grid in half voltage has no other type of capacitive compensation because the distribution grids have short distances for the transport of energy, voltage levels below 34.5 kV and the largest component of conductors are bare wires. It is clarified that for this analysis the reactive power remains almost constant since there is a small decrease in the reactive power losses, although the loads remain consuming the same reactive power. These losses of active power are due to the decrease of the current circulating through the lines with the decrease of the active power delivered by the grid, we can see this in Eq. (5), where it is shown that with a decrease in active power the losses of the reactive power are reduced, but this value is so small in comparison with the variation of active that this reduction in the reactive power delivered by the grid could be considered negligible for this theoretical analysis, however, the exact calculation is detailed below, showing what we have stated before.

(5) $$\Delta Q=\frac{P^{2}+Q^{2}}{V^{2}}X$$

Where, ΔQ are the reactive power losses on the lines, P is the active power delivered by the grid, Q is the reactive power delivered by the grid, V is the line voltage and X is the inductive reactance of the distribution line.

Then the resulting power factor can be calculated as shown in (6).

(6) $$PF=\text{cos}\left(\tan^{-1}\left(\frac{Q-\Delta Q}{P}\right)\right)$$

**Fig. 8:**
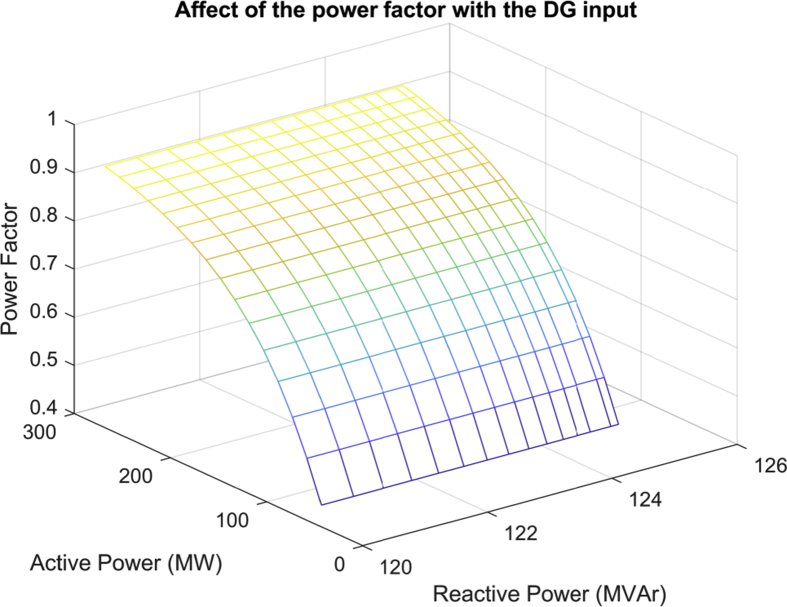
Affectation of the power factor with the reduction of the active power delivered by the grid.

Fig. 8 shows the variation in the power factor for different active power compensations, also considering the reduction of reactive power losses for each decrease of the active in the same scenario of the IEEE distribution system of 30 bars, with a total demand for the active and reactive power load of 283.4 MW y 126.2 MVAr [10].

### 2.11 Mathematical formulation

The reactive power compensation has been analyzed mainly as an optimization problem restricted to a single objective, which would provide a single optimal solution with a priority approach based on the adequate selection of capacity and location of capacitor banks. For this study the objective function is defined as a linear combination of several factors, such as investment costs, power factor improvement and reduction of distribution energy losses, subject to operational limitations such as reliability and stability in the profiles voltage.

As an optimization model, a multi-criteria technique is proposed, referring to the analysis of a set of n decision variables in a distribution system with a set of objective functions k for optimization, and a set of restrictions s[102]. The objective functions and restrictions are functions of the decision variables. This can be expressed as:

(7) F(x)=[F1(x),F2(x),…,Fk(x)]

(8) e(x)=[e1(x),e2(x),…,es(x)]≥0

(9) Wherex=[x1,x2,…,xn]εX

(10) y=[y1,y2,…,yk]εY

x is known as the decision vector while y will be the target vector. X denotes the feasible space of decision and the objective space is denoted by Y. In this case the optimization could mean minimizing or maximizing the variables according to the desired objectives. The set of restrictions e(x)≥0 determines the set of feasible solutions for X, and the set of feasible target vectors Y. From this it can be deduced that the set of solutions produces a target vector y, where all x must satisfy the set of restrictions e(x)≥0. The optimization problem is to find the x which has the “best” F(x).

For the implementation of this technique, it is necessary to determine precisely the decision criteria and their scales of quantifiable measures, for the construction of the eligible set, conformed by the alternatives with their evaluations for each criterion. The criteria must be all of the same type, whether qualitative or quantitative. Finally, the decision matrix is established to choose the optimal solution.

**Fig. 9:**
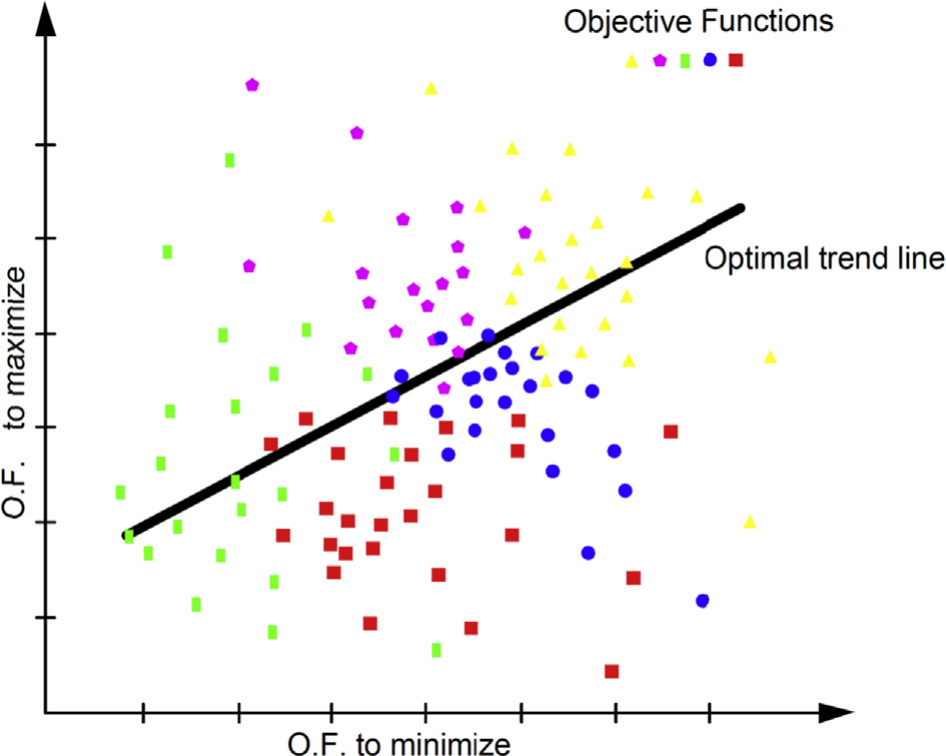
Graphic representation of the optimization model for multiple objective functions.

Fig. 9 visually shows the optimization model for multiple objective functions, where it can be seen that the best individual solutions are those close to the optimum general trend line.

In general, there is no better solution, but a set of solutions, where none of them can be considered better than the others, if the goal is that all objectives are considered at the same time [102, 103] This is because there could be conflicts between the different objectives that form the optimization problem, since the criteria used to observe and define simultaneous decision alternatives are several and of different types.

The majority of authors, who have addressed the topic of optimization of reactive power flows, have focused their analysis on optimization problems with a single objective function, however, in the state of the art, it has been shown that this is a much more complex problem in which the incidence of all variables must be analyzed and current real scenarios with active generation points must be considered. In this mathematical problem, as in most optimization problems with several objective functions, there are some variables that depending on their objectives may conflict with the search for the optimal solution.

For the mathematical formulation of each of the objective functions shown below, some general restrictions will be taken into account:

1- The cost of the compensating devices will be the same in all nodes of the analysis system.
2- Load will be modeled as a constant power, analyzing the worst scenario where demand is maximum.

FO1: *Cost of reactive power compensating devices* [103,104]*.*

(11) $$FO1=\sum_{i=1}^{n}C\left|Q_{i}\right|\geq 0$$

Where:

C is the cost per kVAr installed in the compensating device. This value considers the cost of the device, installation costs and maintenance costs.
$Q_{i}$ is the value of the compensation in kVAr in the node i.
n is the number of nodes in the system.

FO1 is subject to the following restrictions [103]:

1- (12) $$C=\{\begin{array}{c} a\;si\;0\leq Q_{i}\leq Q_{m}\\ b\;si-Q_{m}\leq Q_{i}\leq 0 \end{array}$$
  where $Q_{m}$ is the absolute value in kVAr of the highest possible compensation in a node and (a,b) is the cost per kVAr of the compensating device. General equation of cost value for inductive and capacitive compensation.
2- FO1<máx{FO1}, where FO1 is the investment cost required for the optimization and máx{FO1} is the total investment cost for the complete compensation of the system (trivial solution to the problem).

FO1 is a function to be minimized.

FO2: *Minimization of active power losses.*

(13) $$FO2=\sum_{i=1}^{n}\left(Pg_{i}\right)-\sum_{i=1}^{n}\left(Pc_{i}\right)\geq 0,$$

Where $Pg_{i}$ is the active power generated at the node i in kW and $Pc_{i}$ is the active power in kW demanded by each load at the node i which is connected, being FO2 the total active losses in the system in kW.

FO2 is a function to be minimized.

FO3: *Improvement of the Power Factor (PF).*

  (14) $$FO3=Ø=\tan^{-1}\frac{\sum_{i=1}^{n}Qc_{i}}{\sum_{i=1}^{n}Pc_{i}}$$

Where:

Ø is the angle of the power factor of the system.
$Pc_{i}$ is the active power in kW demanded by each load at the node i.
$Qc_{i}$ is the reactive power in kVAr demanded for each load at the nodei.
FO3 is a function to be minimized.

FO4, FO5: *Objective functions associated with the improvement of voltage profiles.*

FO4: *Average deviation of the voltage in the system.*

(15) $$FO4=\frac{\sum_{i=1}^{n}\left|Vd_{i}-V_{i}\right|}{n}\geq 0$$

Where:

n is the number of nodes in the system
$V_{i}$ is the voltage at bar i in P.U. (per unit)
$Vd_{i}$ is the desired voltage at bar i in P.U.
FO4 is a function to be minimized.

FO5: *Maximum value of voltage deviation.*

This function represents the maximum voltage deviation that can exist in the system to be analyzed.

(16) $$FO5=\underset{1\leq i\leq n}{\max}\left(\left|Vd_{i}-V_{i}\right|\right)\geq 0$$

Where:

n is the number of nodes in the system
$V_{i}$ is the voltage at bar i in P.U. (per unit)
$Vd_{i}$ is the desired voltage at bar i in P.U.
FO5 is a function to be minimized.

FO6: *Total harmonic distortion index (THD)* [28, 32]*.*

(17) $$FO6=THDi\%=100*\frac{\sqrt{\sum_{i=1}^{H}{\left(V_{i},h\right)}^{2}}}{V_{i},1},$$

Where:

$V_{i},h$ Is the voltage component corresponding to the harmonic h at the node i.
$V_{i},1$ is the fundamental component of the voltage (1st harmonic) at node i.
H is the maximum harmonic order to be taken into account in the calculation.
FO6 is a function to be minimized.

The proposed objective functions were accommodated in such a way that they all apply to the optimization problem as a function to be minimized, so as to be able to form the objective vectors to be minimized to solve the proposed optimization problem, being each eligible vector as follows:

(18) MinimizeF=[FO1,FO2,FO3,FO4,FO5,FO6]

Where FO1,FO2,FO3,FO4,FO5,FO6 are each of the objective functions that were previously defined.

In order to obtain a result depending on the sizing and location of the compensating device, it is necessary to establish the decision matrix as shown in Eq. (19), which will have m number of rows that will describe the number of eligible alternatives that meet the criteria of being different, exclusive and exhaustive and that define the different sizes and locations of the compensating devices in the different nodes of the system. The six columns of this decision matrix show the quantitative criteria that are defined by the variables analyzed as objective functions. According to the established decision criteria, the optimum option is selected by first discarding all solutions that are inferior to any other solution.

(19)
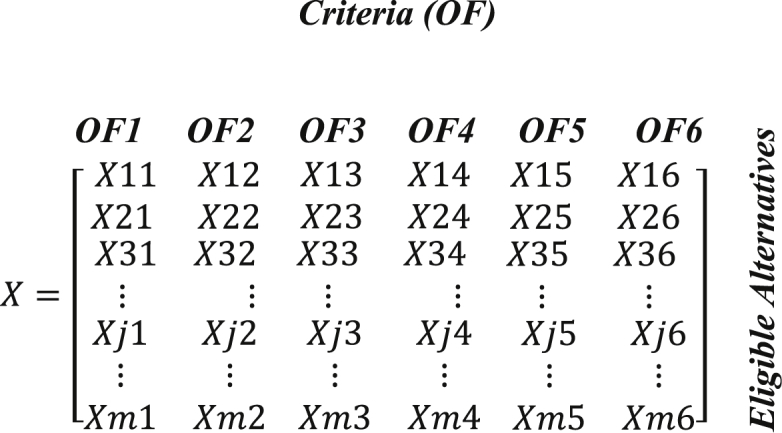


The solution to this optimization problem is to find the best vector X of the set of eligible options, determined by the decision criteria established by the objective vectors. For the problem under analysis, the alternative of location and dimensioning of the reactive power compensating devices would be found.

It is important to take into account that the selection of the capacity of the compensating device must be considered in the analysis as a discrete variable, defined by a vector with the nominal capacities available for distribution grids [67, 93].

### 2.12 Future work

In part II of this research, a case study will be analyzed with the implementation of the proposed mathematical model, where the expected theoretical results will be validated. In addition, the metrics and individual calculations of each of the variables will be analyzed, together with simulations of the study circuit with the location and dimensioning of the compensating devices obtained by the implementation of the heuristics.

As future work, it is also suggested to propose a heuristic method able to find the optimal solution to a simultaneous compensation of active power and reactive power, fulfilling the criteria of efficiency and quality of the power. This can be achieved through the simultaneous dimensioning and optimal location of distributed photovoltaic solar generation and reactive power compensating elements.

It is also recommended to carry out this investigation with the calculation of location and dimensioning of compensating devices in different demand scenarios. The reactive power compensation must also be analyzed in scenarios of minimum demand, where the voltage profiles could reach values above the maximum limit with the reactive power compensation.

## 3 Conclusions

In the present work, a detailed state of the art has been presented, in order to have a clearer perspective of the incidence of the different parameters and variables involved in the reactive power flows in distribution grids, seeking to increase the energy efficiency and quality in these systems.

The concepts of reactive power compensation were presented through the use of different elements and compensating devices, which aim at increasing the efficiency, quality and reliability of the electricity supply in distribution grids.

Through the study of a practical case, the problem of conflict between different variables that exists in the compensation of reactive power with a single objective function was demonstrated.

A thorough bibliographical review on the relevance of the topic and the need for multicriteria treatment to find a truly optimal solution was carried out, taking into account a global approach that assumes all the variables involved in the problem of optimization of reactive power flows.

A multicriterial algorithm for the theoretical solution to the optimization of the reactive power flows is proposed, considering all the variables that intervene in the problem and distribution systems scenarios with distributed resources.

This research demonstrates the importance of the location and optimal dimensioning of compensating devices as an optimization issue that must be solved considering multiple criteria. An error in the location and dimensioning of reactive compensators can lead to the circulation of unwanted reactive power flows, which would affect the variables that determine the efficiency and quality of the energy. It was also demonstrated that distribution grids with distributed resources require a multicriterial analysis due to the conflict that may exist between variables when it is sought to compensate active power from renewable generation specifically from solar photovoltaic sources.

## Declarations

### Author contribution statement

All authors listed have significantly contributed to the development and the writing of this article.

### Funding statement

This research did not receive any specific grant from funding agencies in the public, commercial, or not-for-profit sectors.

### Competing interest statement

The authors declare no conflict of interest.

### Additional information

No additional information is available for this paper.
